# The relationship between spider naevi & de novo arteriovenous malformations in chronic liver disease

**DOI:** 10.1259/bjrcr.20220081

**Published:** 2022-09-12

**Authors:** Sophia G Connor, Paul M Parizel, Victor Wycoco, David A Prentice

**Affiliations:** 1 Department of Neurology, Royal Perth Hospital, Perth, Western Australia, Australia; 2 David Hartley Chair of Radiology, Royal Perth Hospital & University of Western Australia, Perth, Australia; 3 Western Australia National Imaging Facility (WA NIF) Node, Perth, Western Australia; 4 The Neurological Intervention & Imaging Service of Western Australia (NIISWA), Hospital Ave, Nedlands, Western Australia, Australia; 5 The Perron Institute for Translational and Neurological Science, QE II Medical Centre Ralph & Patricia Sarich Neuroscience Building, 8 Verdun St, Nedlands, Western Australia, Australia

## Abstract

We report a patient with decompensated alcoholic liver cirrhosis (Child-Turcotte-Pugh class C) who developed a *de novo* left frontal cerebral AVM and a subcutaneous left temporal scalp spider naevus. Arteriovenous malformations (AVMs) are vascular abnormalities previously thought to be congenital in nature, although new research has revealed the potential for *de novo* AVM formation through a two-hit hypothesis. We propose that the oestrogen-rich environment seen in chronic liver disease could act as the second hit to allow for an angiogenic state favouring *de novo* AVM development. We also postulate that spider naevi are formed through a similar mechanism and may represent early-stage AVMs.

## Introduction

An arteriovenous malformation (AVM) is defined as a tangle of blood vessels, comprising direct connections between arteries and veins, without an interposed a capillary bed, and thus bypassing normal tissues.^1^ AVMs can develop in any organ via angiogenesis, which is the physiological and biochemical process of *de novo* blood vessel formation out of existing blood vessels. This process is mediated by multiple factors, including vascular endothelial growth factor (VEGF), transforming growth factor β (TGF-ß), angio-poietin-1 and nitric oxide (NO) synthase.^2^ It is currently believed that angiogenesis ceases after birth and can subsequently be reactivated only under certain physiological circumstances (*e.g.,* chronic hypoxia), by certain hormones (*e.g.,* oestrogen), or by angiogenic stimulators released by certain tumour cells.^3^

While AVMs have been most often regarded as congenital malformations of the cerebral vasculature, there is an increasing body of literature demonstrating that AVMs are unlikely to be purely congenital in nature, with one theory proposing a “second-hit hypothesis”.^4–6^ The second-hit or two-hit hypothesis suggests that an individual may have an underlying genetic or acquired propensity to develop AVMs,^6,7^ whereby certain insults (such as alcohol-induced hepatic cirrhosis in this report) act to create an angiogenic environment allowing for AVM formation. Pathologies associated with the formation of *de novo* AVMs include, in decreasing order of frequency; other vascular malformations, haemorrhagic stroke, seizures, brain tumours, ischaemic stroke, Moya-moya disease, traumatic brain injury, genetic syndromes, inflammatory diseases and liver cirrhosis.^8^ There have been two cases linking hepatic cirrhosis and AVMs,^4–6^ and two cases of spontaneous AVM resolution post liver transplant.^9,10^ A recent report has also described cerebral arterio-venous shunting as cause of stroke in a patient with advanced liver disease.^11^

We report on the *de novo* formation of a left-sided scalp spider naevus and a cerebral AVM in a patient with end-stage liver cirrhosis. We explore the potential link between chronic liver disease-induced angiogenesis, spider naevi and AVMs. Written informed consent for the case to be published (incl. images, case history and data) was obtained from the patient and the patient’s next of kin.

## Clinical Presentation, Investigations and Differential Diagnoses

A 38-year-old female with known alcoholic liver cirrhosis (Child-Turcotte-Pugh class C, decompensated liver disease) presented to the emergency department after a witnessed generalised tonic-clonic seizure secondary to alcohol withdrawal. She had suffered a previous seizure during pregnancy, which had been managed with carbamazepine for 12 months. Her liver cirrhosis had previously been complicated by ascites, coagulopathy, thrombocytopenia, oesophageal varices and multiple episodes of hepatic encephalopathy. Clinical examination showed a jaundiced patient with palpable hepatomegaly, palmar erythema and multiple spider naevi across her torso. Additionally, she had one prominent subcutaneous spider naevus in the left temporal pre-auricular scalp region measuring 1.0 × 1.6 cm in diameter. She had no previous history of head injury. Neurological examination was normal, aside from mild nystagmus on right lateral gaze. EEG showed no epileptiform activity.

Abdominal ultrasound examination revealed portal hypertension, reversal of portal venous flow, recanalisation of the umbilical vein and splenomegaly. She had no ascites at this time, and her vitals were otherwise unremarkable. Liver function tests revealed a bilirubin 243 µmol l^−1^ (normal range: 3–22), alanine transaminase 73 IU l^−1^ (normal range: 0–50), alkaline phosphatase 216 IU l^−1^ (normal range: 40–150), gamma-glutamyl transpeptidase 151 IU l^−1^ (normal range: 0–55) and albumin 26 g l^−1^ (normal range: 32–45). Her platelets 56 × 10^9^  l^−1^ (normal range: 150–400) and her international normalised ratio was 1.8.

**Figure 1.**
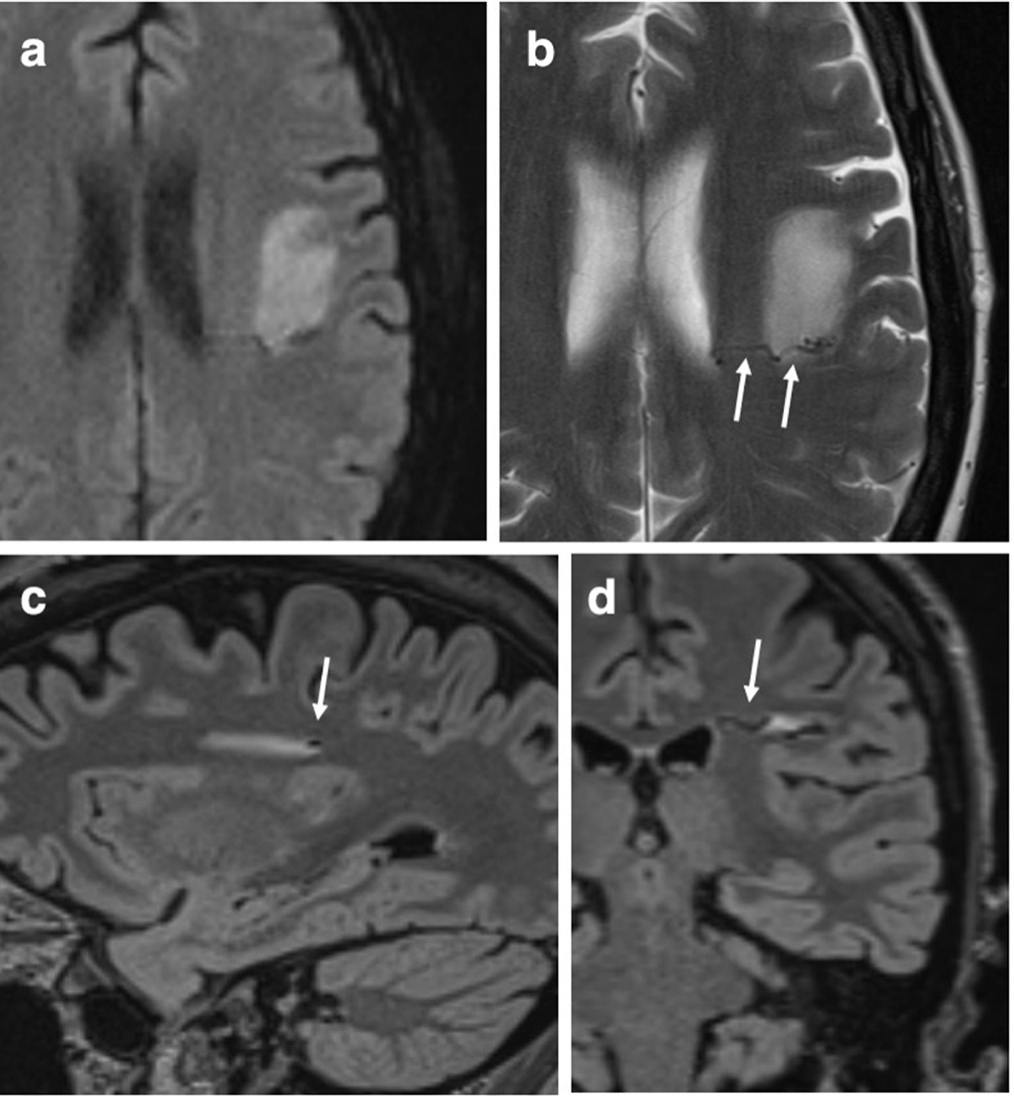
Brain MRI reveals a subacute cerebral infarct in the left frontal white matter as evidenced by increased signal intensity on the diffusion-weighted sequence (**A**) and decreased ADC values (not shown). On the corresponding axial TSE *T*_2_-weighted image (**B**) on the sagittal (**C**) and coronal (**D**) 3D FLAIR images, along the poster margin of the left frontal white matter infarct a serpiginous draining vein is seen, as identified by a flow void (white arrows).

A Chest X-ray was unremarkable. A prior non-contrast CT brain had been performed in 2008, following a motor vehicle accident, and was normal. There were no traumatic lesions, nor was there evidence of an AVM or other parenchymal lesions, within the limitations of a non-contrast CT study. During the current admission, the patient underwent a brain MRI which revealed a focal, sharply defined area of T2 signal hyperintensity and diffusion restriction involving the left frontal subcortical white matter (Figure 1), with a curvilinear flow void along the posterior margin. While no associated dural venous sinus or cortical vein thrombosis was identified, the working diagnosis of venous infarct with associated AVM was made.

**Figure 2.**
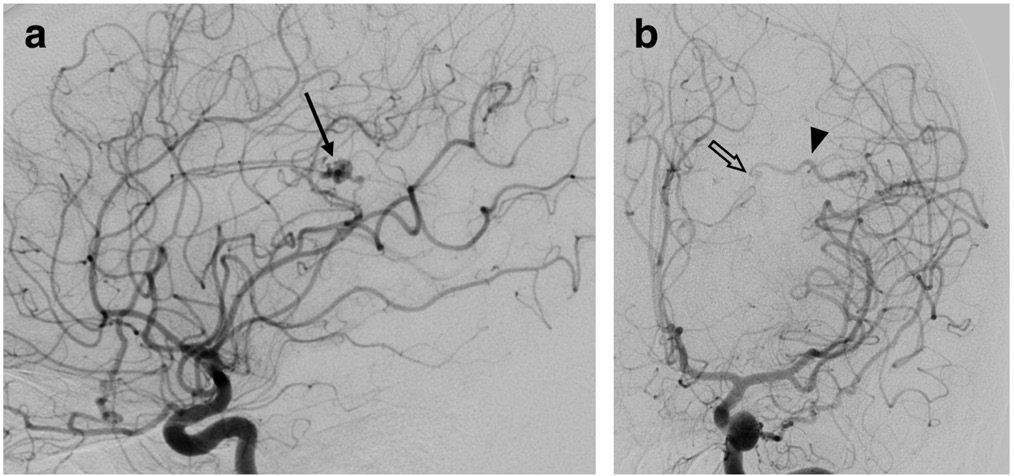
Cerebral digital subtraction angiogram, left internal carotid artery injection with lateral (**A**) and frontal (**B**) projections. A left posterior frontal AVM is identified (arrow), with an irregular calibre vein draining medially (arrowhead), containing segments of venous narrowing (outlined arrow).

**Figure 3.**
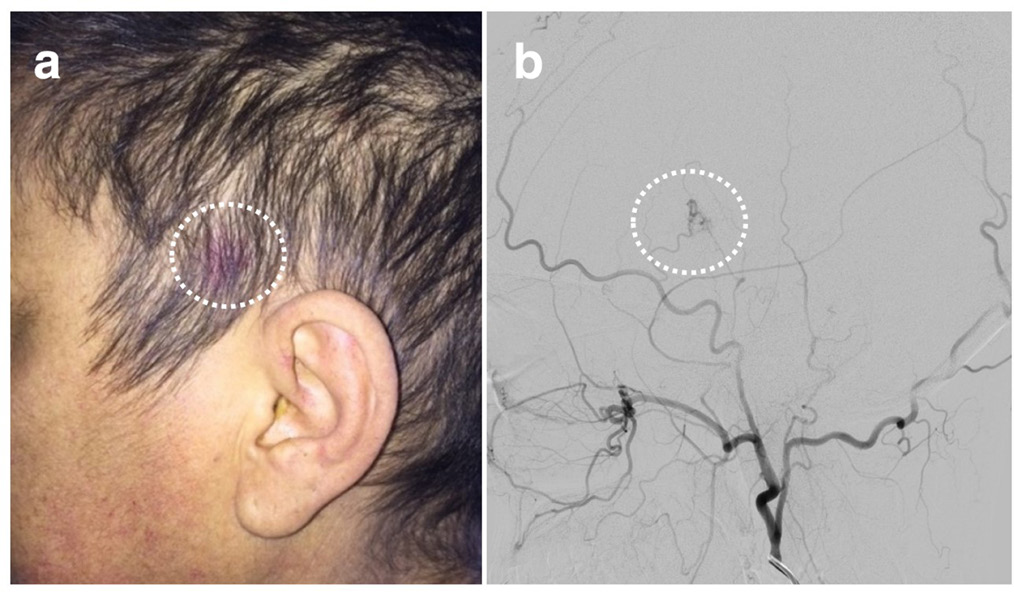
An area of reddish skin discoloration is visible within the hairline in the left temporal region (**A**). The cerebral digital subtraction angiogram, left external carotid artery injection, confirms a small subcutaneous scalp AVM with superficial temporal artery supply in the location of the AVM.

The patient went on to have a catheter cerebral angiogram (Figure 2) which demonstrated an AVM with a 2 mm nidus in the superior margin of the left Sylvian fissure. This was supplied via MCA branches with deep venous drainage into the left internal cerebral vein. The draining vein was irregular in calibre with focal strictures and dilatation, although it drained rapidly on angiography. No associated thrombosis or occluded draining vein was identified. In addition, the spider naevus in the patient’s left preauricular region (Figure 3) was angiographically characterised as another small AVM, with arterial blood supply via the anterior division of the left superficial temporal artery (Figure 3).

## Outcome, Follow-up and Discussion

**Figure 4.**
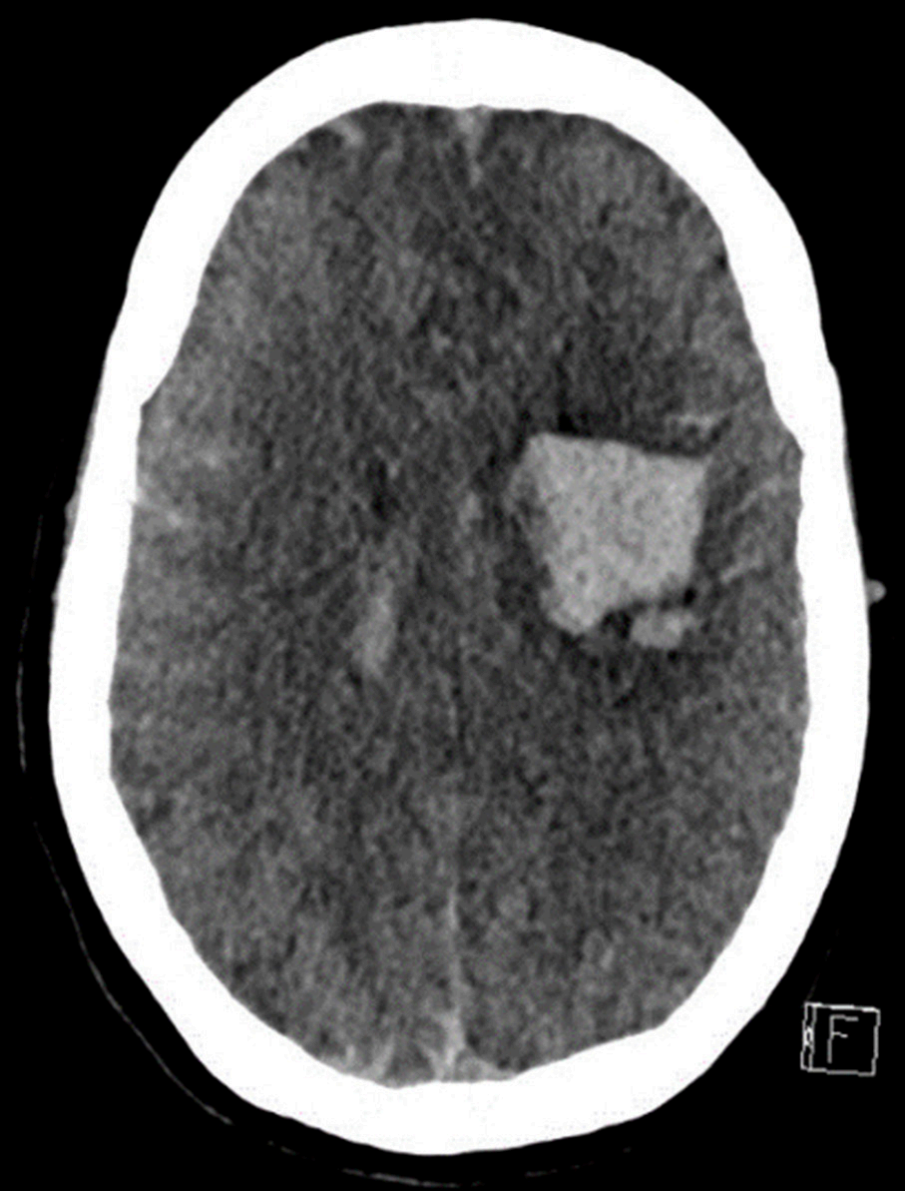
There is an acute left frontal parenchymal haemorrhage, which presents a similar distribution to the area of diffusion restriction identified on MRI in 2016.

The patient did not attend subsequent outpatient neurology clinic appointments and was lost to follow-up. Two years later she presented with an acute left frontal parenchymal haemorrhage likely secondary to the previously described intracranial AVM (Figure 4) and subsequently passed away.

This paper illustrates the formation of a suspected *de novo* AVM, which developed in a patient with chronic liver disease, without any history of conditions associated with AVM development (*i.e.,* hereditary haemorrhagic telangiectasia, Moyamoya disease, venous sinus thrombosis, stroke and other acquired or hereditary brain conditions).^6,8^ We hypothesise that the AVM formed according to the two-hit hypothesis with hepatic cirrhosis acting as the second hit. Only one other case of an associated scalp AVM with a cerebral AVM has been reported.^12^

Patients with advanced chronic liver disease are known to develop peripheral systemic vascular malformations. It is tempting to assume that peripheral and cerebral AVMs share a common set of mechanisms and etiologic factors.^8^ Liver cirrhosis is associated with a high cardiac output state caused by systemic vasodilation, resulting from increased production of NO and reduced hepatic metabolism of vasoconstrictors. The mechanism is complex and, as a consequence of increased splanchnic blood flow, leads to portal hypertension. Oestrogen levels are increased in cirrhosis due to reduced hepatic metabolism. Oestrogen is a powerful vasodilator and promoter of angiogenesis as it increases local and systemic VGEF as well as NO production.^13^ The combined effect of increased blood flow and angiogenesis results in blood vessel and arteriovenous shunt formation. This is well documented in hepatopulmonary syndrome with platypnea-orthodeoxia, which results in pulmonary artery vasodilation and shunting in the basal lung fields. Berthelot et al. documented such shunts in post-mortem lungs from cirrhotic patients finding dilated pulmonary vessels, pleural spider telangiectasia and true arteriovenous fistulae.^14^ Systemic AVMs have also been noted in cirrhotic patients.^10^

It is likely that the mechanisms outlined above, leading to the formation of peripheral vascular malformations in cirrhotic patients, are also responsible for the development of cerebral arterio-venous shunts and AVMs. An intermediate stage in the development of a cerebral AVM has been seen in this case report,^11^ where intracranial arterio-venous shunting was found in a patient with liver cirrhosis.

Spider naevi are vascular lesions found just beneath the skin surface, consisting of a central arteriole with outwardly radiating blood vessels in an arachnid-like shape. They are associated with high-oestrogen states such as pregnancy, oral contraceptive use and liver cirrhosis.^15^ This patient’s scalp AVM clinically has the characteristic of a spider naevus; and previous cases of giant spider naevi could actually represent unrecognized peripheral AVMs.^16,17^ If spider naevi were to be classified as an arteriovenous shunt, then similarly, lung portosystemic shunts could be reclassified as lung spider naevi.^14^ There has even been the suggestion to create a new condition called “Hepato-Pulmonary-Cutaneous Syndrome”.^18^

In the patient presented here, the presence of a left temporal scalp AVM and a left-sided cerebral AVM is unlikely to be mere coincidence, as in a previous study describing a similar phenomenon.^12^ A causal link could be created between the presence of spider naevi indicating peripheral angiogenesis as well as shunt formation, and the presence of similar mechanisms within internal organs leading to cerebral AVMs.^19^

In this case history, given that no MRI brain was conducted in 2008 and taking into account the relative lack of sensitivity of non-contrast CT scans, we cannot formally exclude that the patient may have had a congenital AVM. However, there appears to be good circumstantial evidence for *de novo* formation given the scalp AVM, interval progression of liver disease and literature documenting reversal of AVMs post liver transplantation. As most cerebral AVMs are detected in adolescence or adults, the question of acquired (*de novo*) or congenital development has yet to be answered.

## Learning points

- Liver cirrhosis is associated with both angiogenesis and vasodilation across multiple organ systems.
- *De novo* AVMs are unlikely to be only congenital in nature and may form through a two-hit hypothesis with cirrhosis acting as the second hit.
- Spider naevi can be considered on the spectrum of high flow vascular malformations that includes AVMs where the presence of peripheral spider naevi could represent internal AVM formation.
- Assessment for AVMs and other vascular malformations should be conducted in patients with liver cirrhosis where their symptoms are unexplained by routine imaging

## References

[1] Dalton A, Dobson G, Prasad M, Mukerji N. De novo intracerebral arteriovenous malformations and a review of the theories of their formation. Br J Neurosurg 2018; 32: 305–11. doi: 10.1080/02688697.2018.1478060 29873271

[2] Leblanc GG, Golanov E, Awad IA, Young WL, BoVMotBNW C. Biology of vascular malformations of the brain. Stroke 2009; 40: e694-702. doi: 10.1161/STROKEAHA.109.563692 19834013 PMC2810509

[3] Jesmin S, Mowa CN, Sultana SN, Mia S, Islam R, Zaedi S, et al. Estrogen receptor alpha and beta are both involved in the cerebral VEGF/akt/NO pathway and cerebral angiogenesis in female mice. Biomed Res 2010; 31: 337–46. doi: 10.2220/biomedres.31.337 21187644

[4] Morales-Valero SF, Bortolotti C, Sturiale C, Sturiale CL, Lanzino G. Are parenchymal avms congenital lesions? Neurosurg Focus 2014; 37(3): E2. doi: 10.3171/2014.6.FOCUS14234 25175439

[5] Lasjaunias P. A revised concept of the congenital nature of cerebral arteriovenous malformations. Interv Neuroradiol 1997; 3: 275–81. doi: 10.1177/159101999700300401 20678357

[6] Gondar R, El Rahal A, Kulcsár Z, Schaller K, Momjian S. Spontaneous appearance of de novo intracranial arteriovenous malformation in hepatic cirrhosis. Neurochirurgie 2019; 65: 393–96: S0028-3770(19)30236-X. doi: 10.1016/j.neuchi.2019.09.021 31605684

[7] Jeffree RL, Stoodley MA. Postnatal development of arteriovenous malformations. Pediatr Neurosurg 2009; 45: 296–304. doi: 10.1159/000235604 19690446

[8] Florian IA, Beni L, Moisoiu V, Timis TL, Florian IS, Balașa A, et al. “De novo” brain avms-hypotheses for development and a systematic review of reported cases. Medicina (Kaunas) 2021; 57(3): 201. doi: 10.3390/medicina57030201 33652628 PMC7996785

[9] Shimoda Y, Kuroda S, Kashiwazaki D, Asano T, Yamashita K, Taniguchi M, et al. Spontaneous disappearance of intracranial arteriovenous malformation after living-donor liver transplantation: a case report. No Shinkei Geka 2011; 39: 589–94. 21628738

[10] Alcolado R, Bowry J, Winwood PJ, Loehry CA. Systemic arteriovenous malformations: a feature of advanced liver disease. Gut 1994; 35: 1145–47. doi: 10.1136/gut.35.8.1145 7926922 PMC1375073

[11] Younce JR, Cross DT 3rd, Goyal MS, Lee J-M. Multifocal stroke with proliferation of small cerebral arteries in hepatopulmonary syndrome. Neurol Clin Pract 2018; 8: e15–17. doi: 10.1212/CPJ.0000000000000454 30105173 PMC6075986

[12] Lanzino G, Passacantilli E, Lemole GM Jr, McDougall C, Spetzler RF. Scalp arteriovenous malformation draining into the superior sagittal sinus associated with an intracranial arteriovenous malformation: just a coincidence? case report. Neurosurgery 2003; 52: 440–43. doi: 10.1227/01.neu.0000043934.85424.a2 12535376

[13] Losordo DW, Isner JM. Estrogen and angiogenesis: a review. Arterioscler Thromb Vasc Biol 2001; 21: 6–12. doi: 10.1161/01.atv.21.1.6 11145928

[14] Berthelot P, Walker JG, Sherlock S, Reid L. Arterial changes in the lungs in cirrhosis of the liver--lung spider nevi. N Engl J Med 1966; 274: 291–98. doi: 10.1056/NEJM196602102740601 5903210

[15] Li H, Wang R, Méndez-Sánchez N, Peng Y, Guo X, Qi X. Impact of spider nevus and subcutaneous collateral vessel of chest/abdominal wall on outcomes of liver cirrhosis. Arch Med Sci 2019; 15: 434–48. doi: 10.5114/aoms.2018.74788 30899297 PMC6425211

[16] Sood A, Gupta R, Midha V. A giant spider nevus in A patient of hepatitis C-related liver cirrhosis: A rare presentation. Int J Appl Basic Med Res 2015; 5: 206–7. doi: 10.4103/2229-516X.165373 26539373 PMC4606583

[17] Hane H, Yokota K, Kono M, Muro Y, Akiyama M. Extraordinarily large, giant spider angioma in an alcoholic cirrhotic patient. Int J Dermatol 2014; 53: e119-21. doi: 10.1111/j.1365-4632.2012.05548.x 23451770

[18] Capodicasa E, De Bellis F, Muscat C. The hepato-pulmonary-cutaneous syndrome: description of a case and suggestion of a unifying hypothesis. Case Rep Gastroenterol 2010; 4: 273–78. doi: 10.1159/000319502 21373385 PMC3047757

[19] Alegre-Sánchez A, Bernárdez C, Fonda-Pascual P, Moreno-Arrones OM, López-Gutiérrez JC, Jaén-Olasolo P, et al. Videodermoscopy and doppler-ultrasound in spider naevi: towards a new classification? J Eur Acad Dermatol Venereol 2018; 32: 156–59. doi: 10.1111/jdv.14602 28960458

